# Prehospital blood gas analyses in acute patients treated by a ground-based physician-manned emergency unit: a cohort study

**DOI:** 10.1186/s13049-023-01170-1

**Published:** 2023-12-19

**Authors:** Louise Houlberg Walther, Annmarie Touborg Lassen, Christian Backer Mogensen, Erika Frischknecht Christensen, Søren Mikkelsen

**Affiliations:** 1 The Prehospital Research Unit, Region of Southern Denmark, Odense University Hospital, Odense, Denmark; 2 Department of Regional Health Research, University of Southern Denmark, Odense, Denmark; 3 Department of Emergency Medicine, Odense University Hospital, Odense, Denmark; 4 Emergency Medicine Research Unit, Hospital Soenderjylland, University Hospital of Southern Denmark, Aabenraa, Denmark; 5 Centre for Prehospital and Emergency Research, Aalborg University Hospital and Institute of Clinical Medicine, Aalborg University, Aalborg, Denmark; 6 Department of Emergency and Trauma Care, Center for Internal Medicine and Emergency Care, Aalborg University Hospital, Aalborg, Denmark

**Keywords:** Prehospital, Emergency medical services, Point-of-care, Blood gas, Lactate, pH, CO_2_, Carbon dioxid

## Abstract

**Background:**

The prehospital patients treated by ambulances and mobile emergency care units (MECU) are potentially critically ill or injured. Knowing the risks of serious outcomes in these patients is important for guiding their treatment. Some settings allow for prehospital arterial blood gas analyses. This study aimed to assess the outcomes of prehospital patients in relation to their prehospitally measured lactate, pH, and CO_2_ levels. The primary outcome was 7-day mortality.

**Methods:**

This register-based cohort study included patients with one or more prehospital blood gas analyses during their prehospital treatment by a physician-manned MECU, from January 2015 to December 2018. The blood samples were analyzed on an ABL90 Flex analyzer. Absolute values with percentages and odds ratios (OR) with 95% confidence intervals (CI) were calculated for the primary and secondary outcomes within prespecified subgroups.

**Results:**

The study included 745 patients, with an overall 7-day mortality rate of 20.0%.

**Lactate* level*:**

The 7-day mortality rates were 11.5% in patients with normal lactate levels (< 2.0 mmol/L), 14.4% with intermediate lactate levels (2.0–3.9 mmol/L), and 33.0% with high lactate levels (≥ 4.0 mmol/L). This corresponded to an OR of 1.30 (95% CI: 0.75–2.24) in the intermediate lactate group (2.0–3.9 mmol/L) and an OR of 3.77 (95% CI: 2.44–5.85) in the high lactate group (≥ 4.0 mmol/L), compared to the reference group with normal lactate.

***pH *level:**

The ORs of 7-day mortality rates were 4.82 (95% CI: 3.00–7.75) in patients with blood pH of < 7.35 and 1.33 (95% CI: 0.65–2.72) in patients with blood pH > 7.45, compared to the reference group with normal pH (7.35–7.45).

**CO_2_* level*:**

The ORs of 7-day mortality rates were 2.54 (95% CI: 1.45–4.46) in patients with blood CO_2_ of < 4.3 kPa and 2.62 (95% CI: 1.70–4.03) in patients with blood CO_2_ > 6.0 kPa, compared to the reference group with normal CO_2_ (4.3–6.0 kPa).

**Conclusions:**

This study found a strong correlation between increasing 7-day mortality rates and high blood lactate levels, low levels of pH, and abnormal CO_2_ blood levels, in prehospital patients undergoing prehospital blood analysis.

## Introduction

The patients in need of acute prehospital care comprise a large, inhomogeneous population, ranging from patients with minor injuries to the very sickest of patients. In some settings, advanced mobile emergency care units (MECU) may be available for the most sick and injured patients to supplement the dispatched ambulances. The prehospital services have a range of treatments and diagnostic tools available but their use must be tailored to each patient’s needs. While treatment can be postponed until arrival at the hospital for the healthiest patients, the severely injured or very ill patients’ prognoses can be improved by initiating early and proper prehospital treatments like endotracheal intubation or administration of antibiotics.[1–4].

Prehospital care only contains a few clinical tools compared to the in-hospital standards. Point-of-care (POC) technologies like electrocardiography, measurement of blood glucose, and ultrasonography are now implemented in many prehospital services – some even for many years [5–7]. The use of POC arterial blood gas analysis, however, is only documented in a few prehospital settings. Arterial blood gas analysis is thus available in a specialized critical care aeromedical team in Canada and a ground-based physician-manned MECU in Denmark [8, 9]. In 2019, a randomized clinical study showed that prehospital physicians felt more certain of the patients’ initial diagnoses after reviewing the patients’ blood gas results. Additionally, access to the analysis significantly increased the number of prehospital critical interventions [10].

The POC blood gas analyzer used in that study [10] measured lactate, pH, and pCO2 among other relevant readings such as electrolytes and blood glucose. Blood lactate is a well-known measurement variable when managing critically ill patients within the hospital [11]. The prehospital use of blood lactate measurements is increasing and the patients’ lactate levels are associated with patients’ prognoses in both trauma, medical, and heart diseases [12–15]. Very few studies have investigated the use of prehospital POC analyses of blood pH levels and pCO2 [16]. These two analyses may also assist the prehospital personnel in patient triage.

We conducted this cohort study to gain knowledge about the risk stratification related to prehospital patients’ POC blood analysis results. We focused on the prehospitally measured blood lactate, pH, and pCO2. The primary endpoint was short-term mortality at day 7. Secondary endpoints were 30-day mortality, admission to the hospital, admission to the Intensive Care Unit (ICU), the use of mechanical ventilation, vasopressor treatment, and acute operations.

## Methods

### Study design and setting

This was a register-based cohort study on the usefulness of prehospital blood gas analyses in risk stratification. The study setting was a mixed rural/urban area in Denmark in the catchment area of the MECU located in Odense. The unit consists of a physician specialized in anesthesiology and an emergency medical technician with special training. The MECU covers an area of 2500 km2 and aids a population of approximately 260,000 individuals. In approximately 26% of the cases, the MECU is dispatched as a supplemental resource to the dispatched ambulances. Only around 15% of the patients are diagnosed within the ICD-10 diagnostic chapter concerning injuries. Predominantly, the patients treated by the MECU are diagnosed as having medical illnesses. Following treatment, approximately 10% of the patients are released at the scene with no need for further treatment.[17] The median response time for the MECU is 8 min [17] and the median time from first patient contact to arrival at the hospital is 35.5 min.[18].

### Study population

Patients were eligible for inclusion in this study if they were attended to and treated by the MECU and they had their blood samples successfully analyzed by the ABL90 placed in the MECU from January 2015 until December 2018. Blood sampling was carried out at the discretion of the anesthesiologist operating the MECU.

The patients were identified by using the unique Danish Civil Personal Register (CPR) number [19]. Exclusion criteria were patients without a Danish CPR number and other unidentifiable patients.

### Study material

Since 2013 the MECU in Odense has had an ABL90™ FLEX® (Radiometer, Brønshøj, Denmark) (ABL) installed in the car as a standard stationary point-of-care device. The performance of this smaller device equals larger stationary analyzers, for example, the ABL835™ FLEX® (Radiometer, Denmark) and the GEM 4000® (Werfer, Austria) [20, 21]. The ABL in the MECU has been slightly modified from its original design to withstand the gravitational forces that rapid driving and fast alterations in speed in the MECU entail. The ABL is placed in a specially designed cushioned cradle in the passenger seat of the car [8]. The device and an additional backup device are subject to regular maintenance and calibration carried out at the Department of Clinical Biochemistry at Odense University Hospital. The device’s measurements are calibrated against an in-hospital master device, ABL 800 FLEX (Radiometer, Brønshøj, Denmark). Both the physician and the specialized emergency medical technician in the MECU are trained and capable of operating the ABL. Upon obtaining the blood sample, the sample is immediately injected into the analyzer. The following measurements are available after 35 s: pH, pO_2_, pCO_2_, O_2_Hb, and sO_2_, level of HCO_3_^−^, and base excess, blood glucose level, lactate, bilirubin, hemoglobin, met hemoglobin, and CO-hemoglobin as well as the electrolytes Ca^2+^, K^+^, and Na^+^.

The MECU physician usually draws arterial blood samples. However, venous blood samples may also be analyzed, most often in conjunction with the placement of an intravenous line.

### Outcomes

#### The primary outcome was 7-day mortality

Secondary outcomes were 30-day mortality, admission to the hospital, admission to the ICU, the use of mechanical ventilation, vasopressor treatment, and acute operations within 24 h of the hospital admission.

### Data management

Data on ABL analysis results are automatically stored in a central in-hospital computer in the Department of Clinical Biochemistry where maintaining and calibrating the ABL device also takes place. The ABL analysis results from the study period were transferred to an Excel spreadsheet (MS Excel, Microsoft Corp., Redmond, WA, USA) including the time stamp data. The CPR number of the patient was also transferred if the MECU personnel had entered this manually in the ABL device during patient treatment. If the CPR number was missing, the study investigator manually had to cross-link the time of the ABL analysis to the time of a MECU dispatch in the MECU database and then enter the CPR number into Excel. An ABL analysis result was excluded from the study if the case could not be unequivocally linked to one specific patient according to the procedure described above. Such cases could be, for example, a traffic accident with more than one patient and no coincident registration of CPR number in the ABL 90 file system. All CPR numbers entered by the MECU personnel were also checked by cross-linking the time stamp on the device and the time stamp of the MECU dispatch. In the case of several ABL analyses in one patient, the first valid analysis was used. An analysis was considered valid if the ABL displayed a value in the different parameters no matter what value was displayed.

Data on patients’ outcomes was obtained from The Danish Central Registries and linked to the study participants’ CPR numbers on a highly secured server, where the investigators could only gain access to pseudonymized data.

### Statistical analyses

Statistical analyses were performed using STATA® 17.0 (StataCorp, LLC, College Station, Texas, USA). The distributions of the categorical variables are presented as absolute numbers and percentages. Continuous variables are presented as mean ± standard deviation or median and interquartile range (IQR), respectively.

Univariate analyses were carried out using logistic regression models with both categorical and continuous variables. Results are presented as crude/adjusted odds ratios (OR) with their corresponding 95% confidence interval (CI). The ORs were adjusted for the confounders: Age, sex, and Charlson’s Comorbidity Index (CCI). We have used the updated version of the CCI calculation from 2011 [22]. The CCI scores the severity of patients’ comorbidities within 12 different domains and has a total score range between 0–24. The score is categorized into three grades: mild (CCI score 0–2), moderate (CCI score 3–4) and severe (CCI score ≥ 5).

We did not expect the correlation between 7-day mortality and pH level to be linear as the normal value of pH lies in the middle of the range. The same applies to the correlation between 7-day mortality and CO_2_ level. Therefore, we present these correlations using splines. Subsequently, ORs at different pH/CO_2_ levels with 95% CIs were calculated from the fitted splines.

Post hoc multivariate analyses of the interaction between lactate, pH level, and CO_2_ level were tested using logistic regressions and likelihood ratio tests. The risk of 7-day mortality was also calculated post hoc on data stratified on both pH and CO_2_ levels to explore if a low CO_2_ level increased the risk of 7-day mortality in case of a low pH level.

### Study approval

The study is a register-based cohort study and approval to receive the patient data was granted by the Danish Patient Safety Authority (Ref. 3–3013-2964/1). Permission to store and confidentially work with the personal patient data was granted by the Southern Region of Denmark (Ref. 19/17002).

## Results

**Fig. 1:**
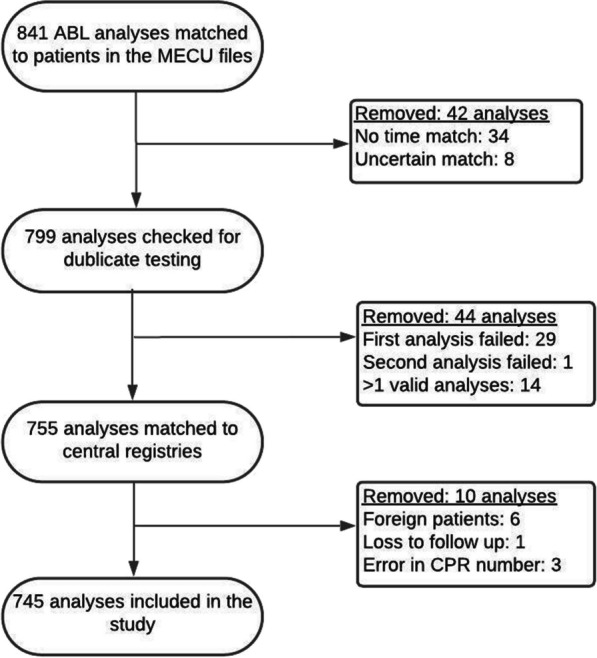
Flow chart of data selection. ABL = ABL90 intern memory files. MECU = Medical Emergency Care Unit. CPR number = Central Personal Registration number

A total of 841 blood gases were drawn in 797 patients from the 1st of January 2015 until the 31st of December 2018, where the MECU treated a total number of 9015 patients (8.8%, 95% CI: 8.3%-9.5%). In 42 cases, the arterial blood gas analysis could not be unequivocally attributed to an identifiable patient. One patient emigrated from Denmark shortly after inclusion in the study and was loss to follow-up. A total of 745 blood gas measurements remained for further analysis. See Fig. 1 for detailed information on data selection.

In 94 cases (12.6%), patients were treated prehospitally without the need for subsequent transportation to the hospital. In 13 of these 94 cases (13.8%), patients were pronounced dead on the scene. The hospital admission rate was 63.9% (95% CI: 60.3–67.3) with a median length of stay at the hospital of 5 days (IQR: 2–9 days). Of all patients treated prehospitally, 27.2% (95% CI: 24.1–30.6%) were admitted to an ICU, 21.6% (95%CI: 18.7–24.7%) received mechanical ventilation, 14.0% (95% CI: 11.6–16.7%) received vasopressor therapy and 11.1% (95% CI: 9.0–13.6%) underwent an early operation within the first 24 h of admission.

The total study population had a 7-day mortality rate of 20.0% (95% CI: 17.2–23.1) with increasing risk at higher ages. The patients had the highest mortality if they were assigned diagnoses within the circulatory system or had infections. Patients’ baseline data and absolute numbers on the primary outcome 7-day mortality are shown in Table 1.

**Table 1:** Demographic data and risk of 7-day mortality in the different strata

	N (%)	7-day mortality (%)
All patients	745	149 (20.0)
Sex (male)	302 (40.5)	83 (18.7)
Age, years*	69 (54;79)	
*Age groups*		
< 18 years	16 (2.1)	0 (0)
18–39 years	85 (11.4)	5 (5.9)
40–59 years	149 (20.0)	12 (8.1)
60–79 years	309 (41.5)	71 (23.0)
≥ 80 years	186 (25.0)	61 (32.8)
*Charlson’s comorbidity index*		
0–2	595 (79.9)	118 (19.8)
3–4	100 (13.4)	18 (18.0)
≥ 5	50 (6.7)	13 (26.0)
Final diagnosis group**		
Unclassified symptoms [18]	165 (22.1)	26 (15.8)
Respiratory system [10]	125 (16.8)	18 (14.4)
Injuries [19]	91 (12.2)	6 (6.6)
Circulatory system [9]	85 (11.4)	50 (58.8)
Unclassified causes [21]	71 (9.5)	12 (16.9)
Infection [1]	41 (5.5)	10 (24.4)
Endocrine/metabolic [4]	18 (2.4)	< 4
Digestive system [11]	18 (2.4)	< 4
Nervous system[6]	15 (2.0)	< 4
Other	22 (3.0)	4 (18.2)
No diagnosis***	94 (12.6)	16 (17.0)
*Lactate group (mmol/L)*		
< 2.0	295 (39.6)	34 (11.5)
2.0 – 3.9	180 (24.2)	26 (14.4)
≥ 4.0	270 (36.2)	89 (33.0)
*pH group*		
< 7.35	355 (47.7)	112 (31.5)
7.35 – 7.45	275 (36.9)	24 (8.7)
≥ 7.45	115 (15.4)	13 (11.3)
*pCO*_*2*_* group*		
< 4.3	108 (14.5)	27 (25.0)
4.3 – 6.0	293 (39.3)	34 (11.6)
≥ 7.45	344 (46.2)	88 (25.6)

*Age is presented as the median with 25% and 75% interquartile.** The numbers in brackets are the ICD-10 group number, version 2016.*** Patients treated prehospitally without subsequent transport to the hospital did not receive diagnoses

**Fig. 2:**
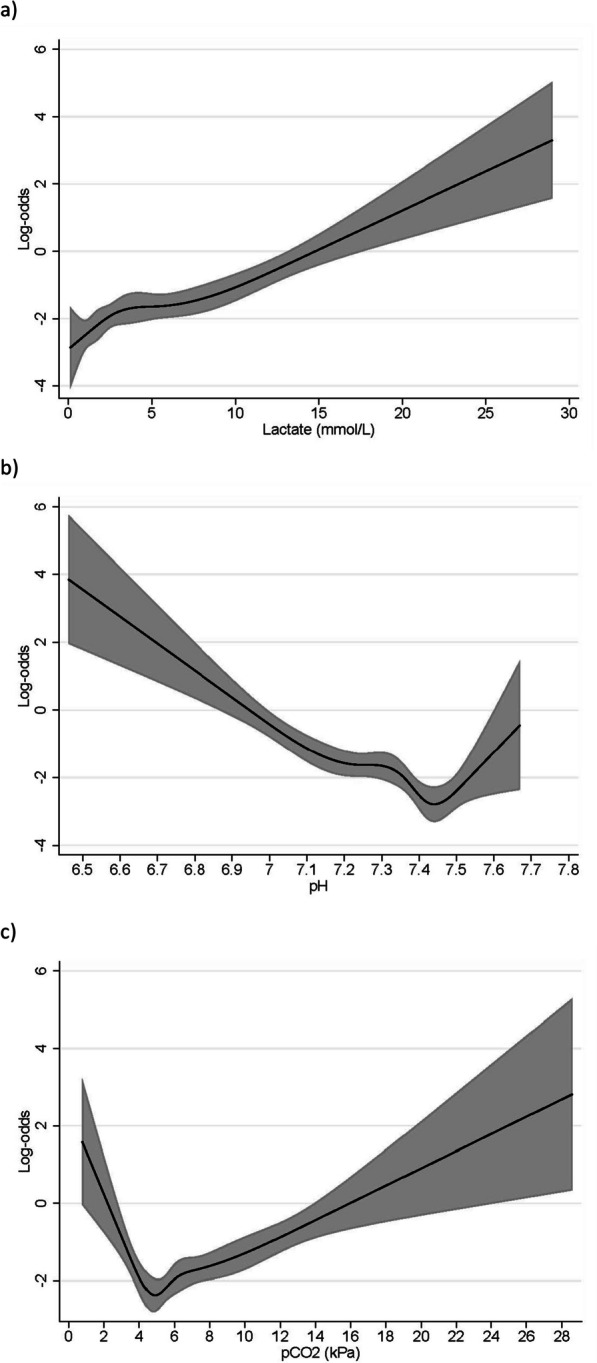
**a**–**c** The correlation between 7-day mortality and levels of lactate, pH, and CO_2_ using splines. Visual illustrations of the correlations between lactate/pH/CO2 levels and 7-day mortality. The light grey areas represent the 95% CI levels

The unadjusted odds ratios (OR) and the ORs adjusted for the confounders: Age, sex, and comorbidity on the risk of 7-day mortality are presented in Table 2. There was a strong and significant correlation between 7-day mortality and increasing levels of lactate, pH levels below the normal range, and both high *and* low levels of CO_2_. The risk of 7-day mortality increased in a linear matter with increasing lactate value as seen in Fig. 2a. As expected, the correlations between pH/CO_2_ and 7-day mortality were not linear, and graphic illustrations of the correlations are shown in Fig. 2b-c. The ORs of 7-day mortality appeared to be lowest at a pH level of approximately 7.43 and a CO_2_ level of approximately 4.6 kPa.

**Table 2:** Odds ratios for the primary outcome 7-day mortality

Outcome	Parameter	Crude Odds Ratio (95% CI)	p Value	Adjusted Odds Ratio (95% CI)	p Value
7-day Mortality	Lactate level (continuous)	1.16 (1.11–1.20)	< 0.0001	1.18 (1.14–1.23)	< 0.0001
	*Lactate group (mmol/L)*				
	< 2.0 (reference)	1		1	
	2.0–3.9	1.30 (0.75–2.24)	0.354	1.37 (0.78–2.42)	0.271
	≥ 4.0	3.77 (2.44–5.85)	< 0.001	3.85 (2.43–6.11)	< 0.001
	*pH group*				
	7.35–7.45 (reference)	1		1	
	< 7.35	4.82 (3.00–7.75)	< 0.001	4.68 (2.87–7.63)	< 0.001
	> 7.45	1.33 (0.65–2.72)	0.430	1.37 (0.66–2.85)	0.398
	*CO*_*2*_* group (kPa)*				
	4.3–6.0 (reference)	1		1	
	< 4.3	2.54 (1.45–4.46)	0.001	2.72 (1.51–4.92)	0.001
	> 6.0	2.62 (1.70–4.03)	< 0.001	2.49 (1.59–3.89)	< 0.001

The adjusted odds ratios are adjusted for the confounders: Age, sex, and Charlson’s comorbidity index

After fitting the splines, specific ORs for 7-day mortality were calculated for different levels of pH and CO_2_. The results are presented in Tables 3 and 4.

**Table 3:** Odds ratios for 7-day mortality at different pH levels, adjusted for confounders: Age, sex, and Charlson’s comorbidity index

pH level	Odds ratio (95% CI)
7.0	8.50 (4.87–14.85)
7.1	4.13 (2.39–7.13)
7.2	2.73 (1.62–4.59)
7.3	2.52 (1.47–4.33)
7.4 (reference)	1
7.5	1.26 (0.74–2.15)
7.6	4.11 (0.90–18.67)

**Table 4:** Odds ratios for 7-day mortality at different levels of CO_2_, adjusted for confounders: Age, sex, and Charlson’s comorbidity index

CO_2_ (kPa)	Odds ratio (95% CI)
2.0	13.26 (3.96–44.41)
3.0	4.29 (2.19–8.42)
4.0	1.54 (1.20–1.97)
5.0 (reference)	1
6.0	1.50 (0.93–2.41)
7.0	1.85 (1.11–3.09)
8.0	2.10 (1.26–3.48)
10.0	2.92 (1.67–5.10)
12.0	4.41 (2.57–7.57)
15.0	8.39 (4.26–16.55)

Table 5 shows the results of the secondary outcomes: 30-day mortality, admission to the Intensive Care Unit (ICU), the use of mechanical ventilation, vasopressor treatment, and acute operations. Overall, an increasing lactate level was correlated strongly and significantly with increasing risk of all secondary outcome parameters. When the lactate levels were divided into 3 groups, only the group with lactate levels ≥ 4 was correlated strongly to 30-day mortality.

**Table 5:** Odds ratios for the secondary outcomes

Outcome	Parameter	Crude odds ratio (95% CI)	*p* Value	Adjusted odds ratio (95% CI)	*p* Value
30-day mortality	Lactate level (continuous)	1.16 (1.11–1.21)	< 0.001	1.20 (1.15–1.25)	< 0.001
	*Lactate group (mmol/L)*				
	< 2.0 (reference)	1		1	
	2.0–3.9	1.20 (0.74–1.95)	0.464	1.28 (0.77–2.13)	0.341
	≥ 4.0	3.65 (2.46–5.40)	< 0.001	3.87 (2.54–5.91)	< 0.001
	*pH group*				
	7.35–7.45 (reference)	1		1	
	< 7.35	3.36 (2.27–4.98)	< 0.001	3.25 (2.15–4.92)	< 0.001
	> 7.45	1.03 (0.56–1.88)	0.925	1.05 (0.56–1.97)	0.880
	*CO*_*2*_* group (kPa)*				
	4.3–6.0 (reference)	1		1	
	< 4.3	2.00 (1.20–3.33)	0.008	2.12 (1.23–3.66)	0.007
	> 6.0	2.26 (1.55–3.30)	< 0.001	2.12 (1.42–3.16)	< 0.001
Intensive care	Lactate level (continuous)	1.13 (1.09–1.17)	< 0.001	1.13 (1.09–1.17)	< 0.001
	*Lactate group*				
	< 2.0 (reference)	1		1	
	2.0–3.9	1.80 (1.14–2.86)	0.012	1.80 (1.14–2.86)	0.012
	≥ 4.0	3.84 (2.58–5.71)	< 0.001	4.02 (2.68–6.02)	< 0.001
	*pH group*				
	7.35–7.45 (reference)	1		1	
	< 7.35	4.43 (2.97–6.60)	< 0.001	4.68 (3.12–7.02)	< 0.001
	> 7.45	0.84 (0.44–1.61)	0.598	0.83 (0.43–1.60)	0.579
	*CO*_*2*_* group*				
	4.3–6.0 (reference)	1		1	
	< 4.3	(0.84–2.53)	0.178	1.43 (0.82–2.48)	0.204
	> 6.0	(2.15–4.58)	< 0.001	3.25 (2.22–4.77)	< 0.001
Mechanical ventilation	Lactate—continuous (mmol/L)	1.13 (1.09–1.17)	< 0.001	1.13 (1.09–1.17)	< 0.001
	*Lactate group*				
	< 2.0 (reference)	1		1	
	2.0–3.9	1.99 (1.19–3.30)	0.008	1.98 (1.19–3.30)	0.008
	≥ 4.0	3.84 (2.48–5.95)	< 0.001	3.99 (2.56–6.22)	< 0.001
	*pH group*				
	7.35–7.45 (reference)	1		1	
	< 7.35	4.44 (2.85–6.92)	< 0.001	4.70 (3.00–7.37)	< 0.001
	> 7.45	0.81 (0.38–1.72)	0.579	0.80 (0.38–1.70)	0.563
	*CO*_*2*_* group (kPa)*				
	4.3–6.0 (reference)	1		1	
	< 4.3	1.47 (0.78–2.77)	0.231	1.45 (0.77–2.74)	0.250
	> 6.0	3.75 (2.45–5.75)	< 0.001	3.94 (2.56–6.07)	< 0.001
Need of vasopressor	Lactate—continuous (mmol/L)	1.13 (1.08–1.17)	< 0.001	1.13 (1.08–1.17)	< 0.001
	*Lactate group*				
	< 2.0 (reference)	1		1	
	2.0–3.9	2.94 (1.53–5.66)	0.001	2.95 (1.53–5.67)	0.001
	≥ 4.0	5.20 (2.92–9.27)	< 0.001	(2.98–9.59)	< 0.001
	*pH group*				
	7.35–7.45 (reference)	1		1	
	< 7.35	3.29 (1.97–5.50)	< 0.001	3.35 (2.00–5.61)	< 0.001
	> 7.45	0.78 (0.32–1.90)	0.590	0.78 (0.32–1.89)	0.585
	*CO*_*2*_* group (kPa)*				
	4.3–6.0 (reference)	1		1	
	< 4.3	1.89 (0.95–3.78)	0.071	1.89 (0.95–3.79)	0.071
	> 6.0	2.79 (1.68–4.61)	< 0.001	2.84 (1.71–4.71)	< 0.001
Urgent operation	Lactate—continuous (mmol/L)	1.09 (1.05–1.14)	< 0.001	1.09 (1.04–1.13)	< 0.001
	*Lactate group*				
	< 2.0 (reference)	1		1	
	2.0–3.9	2.81 (1.46–5.43)	0.002	2.79 (1.45–5.40)	0.002
	≥ 4.0	3.21 (1.76–5.86)	< 0.001	3.03 (1.65–5.57)	< 0.001
	*pH group*				
	7.35–7.45 (reference)	1		1	
	< 7.35	2.47 (1.43–4.27)	0.001	2.51 (1.44–4.37)	0.001
	> 7.45	1.14 (0.50–2.61)	0.749	1.13 (0.49–2.58)	0.773
	*CO*_*2*_* group (kPa)*				
	4.3–6.0 (reference)	1		1	
	< 4.3	1.79 (0.92–3.48)	0.088	1.84 (0.94–3.61)	0.075
	> 6.0	1.39 (0.83–2.33)	0.213	1.50 (0.89–2.54)	0.130

The adjusted odds ratios are adjusted for the confounders: Age, sex, and Charlson’s comorbidity index

Likewise, decreasing pH level was correlated significantly with increasing risk of secondary outcomes, compared to patients with normal levels of pH. There was also a strong correlation between increasing levels of CO_2_ and increasing risk of all secondary outcomes except urgent operations, compared to patients with normal levels of CO_2_. Additionally, a strong correlation was observed between a lower level compared to the normal level of CO_2_ and an increased risk of 30-day mortality.

Post hoc multivariate logistic regression analyses of the interaction between lactate, pH, and CO_2_ levels and 7-day mortality were tested with likelihood ratio tests for possible interactions and showed a *p* = 0.56 for lactate and pH, *p* = 0.62 for lactate and CO_2_, and a *p* = 0.11 for pH and CO_2_. These high p-values did not suggest possible interactions between the three biomarkers in correlation to the 7-day mortality risk.

Post hoc stratified logistic regression analysis showed that in the patients with low pH levels also having CO_2_ levels below normal range, the 7-day mortality risk increased with an OR = 5.13 (95% CI 1.8–14.1, *p* = 0.002) compared to patients with low pH levels and normal CO_2_ levels.

## Discussion

This cohort study found strong evidence of increasing risk of 7-day mortality and 30-day mortality with high blood lactate levels, low pH levels, and abnormal CO_2_ levels in patients undergoing prehospital blood analyses. The need for ICU admission, mechanical ventilation, or need of vasopressor therapy all increased with increasing lactate, lower pH, and higher CO_2_ levels. The study population had a very high overall 7-day mortality rate of 20.0%, confirming this was a critically ill and injured patient group.

The study showed strong evidence of a linear increasing 7-day mortality risk with increasing levels of lactate. A few other studies of prehospital lactate measurements have found comparable results, but they have not investigated an undifferentiated patient population [13–15, 23]. Swan et al. [13] found an OR = 3.76 of in-hospital mortality in 253 medical patients if paramedics measured a prehospital lactate level > 2.5 mmol/L on a handheld lactate meter. Tobias et al. [23] included a larger sample of 673 prehospital non-trauma patients and found an OR = 3.57 of in-hospital death if the patient had a lactate level > 2.0 mmol/L. Guyette et al. [15] investigated 1168 trauma patients and found a strong correlation between prehospital measured high lactate levels and in-hospital mortality with an OR = 1.23.

Few studies have investigated the correlation between prehospital measured lactate levels and outcomes other than mortality. Swan et al. [13] reported an increased risk of ICU admission with increasing prehospital lactate levels in adult medical patients, with a median prehospital lactate level of 3.2 (95% CI: 2.4–5.7) in ICU patients versus a median level of 2.4 mmol/L (95% CI: 1.5–3.6) in non-ICU patients.

The study showed very strong evidence of increasing 7-day mortality risk with pH values below the normal range (pH < 7.35). Even a small decline in pH level to 7.30 shows clear evidence of increased risk with an OR of 2.52 (95% CI 1.47–4.33) and the risk increases with decreasing pH levels. The spline of the correlation between pH level and 7-day mortality indicates an increase in risk with pH values above the normal range (pH > 7.45), but the calculated ORs in Table 3 for these values show no statistical evidence for this correlation. A minority of the patients had pH levels above the normal range (15.4%) causing wide 95% CIs at these pH levels. Especially for pH 7.6, OR was 4.11 (95% CI: 0.90–18.67). A larger sample size may have gained higher power to show evidence of this correlation.

Few studies have evaluated the correlation between prehospitally measured pH levels and mortality. A German study investigated 98 patients suffering from out-of-hospital cardiac arrest and had prehospital blood gases measured by a handheld POC analyzer. Patients surviving to discharge had a median pH level of 7.16 while deceased patients had a median pH level of 7.02 (*p* = 0.072) [16].

A low CO_2_ level was highly correlated to increased 7-day and 30-day mortality but was not correlated to ICU admission, mechanical ventilation, or vasopressor therapy. Low levels of CO_2_ may indicate a larger need for observation than was observed in this study, but we cannot conclude that causality exists.

The ORs presented in Table 4 show very clear evidence of increased 7-day mortality risk already at a slightly decreased level of CO_2_ of 4.0kPa. A CO_2_ level of 3.0 kPa increases the 7-day mortality risk to the same extent as a very elevated CO_2_ value of 12.0 kPa.

We have not been able to find prior studies investigating prehospital measured blood CO_2_ levels, but studies have been published on end-tidal (ET) CO_2_ in the prehospital setting. These studies showed increased mortality risk at low ET CO_2_ levels, but not at high ET CO_2_ levels [24–26]. A prehospital cohort study of 1,088 undifferentiated prehospital patients demonstrated a strong correlation between low ET CO_2_ and mortality with a mean ET CO_2_ of 34mmHg (4.5 kPa) in survivors vs. 25 mmHg (3.3 kPa) in non-survivors, *p* < 0.001. The authors found that abnormal prehospital ET CO_2_ (lower than 32 or higher than 40 mmHg) (4.3 kPa and 5.3 kPa) performs better than other prehospital vital signs like blood pressure and respiratory rate in predicting mortality [24].

We have investigated each of the three analyses’ (lactate, pH, and CO_2_) individual correlations to the outcomes in this study, but these three parameters are physiologically linked to each other. If a patient is hyperventilating, a drop in blood CO_2_ level occurs which, in turn, raises the pH level. Equivalently, a rise in lactate level will often occur together with a drop in pH level, which can be compensated by hyperventilation eventually leading to lower CO_2_ and a rise/normalization of the pH level. This study cannot conclude whether a low CO_2_ level solely correlates to an increased 7-day mortality risk or is merely a compensatory mechanism because of a low pH level. Our post hoc stratified logistic regression analysis indicates that in the case of a low pH level, having a low CO_2_ level still increases the risk of 7-day mortality compared to having a low pH level and a normal level of CO_2_. However, post hoc likelihood ratio tests did not show evidence of interactions between lactate levels, pH levels, or CO_2_ levels on 7-day mortality risk.

### Strengths & limitations

Only a few EMS have reported access to POC blood gas analyses and consequently, reported outcomes are seldom related to lactate, pH, and CO_2_ levels in prehospital cohorts. Only one patient was loss to follow-up. This enables results with good intern validity. In addition, data from the Danish Central registries are considered reliable [27].

The ABL90 Flex is calibrated against a Golden Standard stationary analyzer (ABL800 Flex) and calibrated every eight hours and coinciding with every measurement. Therefore, the equipment used in this study presents reliable blood gas results comparable to the results provided for the in-hospital clinicians.

The study material consists of both arterial and venous blood samples, and levels of lactate, pH, and CO_2_ might vary between sample sites. Prior studies have shown acceptable agreement in measurements taken from different sample sites [28–30]. However, a difference in venous and arterial CO_2_ levels can be present in critically ill patients [31].

The external validity is limited as the patient group can only be considered a convenience sample. The patients were included according to the attending MECU physicians establishing indications for blood gas analysis. No in-hospital or prehospital guidelines exist on when to do a POC blood analysis. We assume that the measurements were only applied to the most seriously ill or injured patients, but they may well have been omitted in patients with an obvious and immediate need for critical interventions–as in patients with cardiac arrests or severely ill septic patients. Comprising only patients from a convenience sample, the study could not demonstrate any benefit of ABG analysis as an additive to the usual diagnostic tools available in the prehospital setting. However, we have previously demonstrated that in our case mix, randomization of patients to have an ABG analysis performed led to an increase in critical interventions in patients with reduced consciousness [10].

It is a further limitation that 42 cases of blood gas analysis could not be unequivocally correlated with an identifiable patient. The missing data from these 42 patients is expected to be missing at random and should not bias the study results.

## Conclusion

This study investigated prehospital risk stratification with POC blood gas analysis in a large population of potentially seriously ill or injured prehospital patients attended to by a MECU physician, who found indication for prehospital POC blood analysis. We found strong evidence that increasing lactate levels, decreasing pH levels, and abnormal levels of CO_2_ correlate to increased 7-day mortality and 30-day mortality. Additionally, Increasing lactate levels, low pH levels, and higher CO_2_ levels were strongly correlated to increased risk of ICU admission, need for mechanical ventilation, and need for vasopressor therapy.

## Data Availability

The dataset generated from the ABL 90 results during the current study is available from the corresponding author upon reasonable request.
